# Make It Short and Easy: Username Complexity Determines Trustworthiness Above and Beyond Objective Reputation

**DOI:** 10.3389/fpsyg.2017.02200

**Published:** 2017-12-19

**Authors:** Rita R. Silva, Nina Chrobot, Eryn Newman, Norbert Schwarz, Sascha Topolinski

**Affiliations:** ^1^Social Cognition Center Cologne, University of Cologne, Cologne, Germany; ^2^Department of Psychology, SWPS University of Social Sciences and Humanities, Warsaw, Poland; ^3^Mind and Society Center, University of Southern California, Los Angeles, CA, United States; ^4^Department of Psychology, University of Southern California, Los Angeles, CA, United States

**Keywords:** name pronounceability, name length, fluency, trustworthiness, reputation

## Abstract

Can the mere name of a seller determine his trustworthiness in the eye of the consumer? In 10 studies (total *N* = 608) we explored username complexity and trustworthiness of eBay seller profiles. Name complexity was manipulated through variations in username pronounceability and length. These dimensions had strong, independent effects on trustworthiness, with sellers with easy-to-pronounce or short usernames being rated as more trustworthy than sellers with difficult-to-pronounce or long usernames, respectively. Both effects were repeatedly found even when objective information about seller reputation was available. We hypothesized the effect of name complexity on trustworthiness to be based on the experience of high vs. low processing fluency, with little awareness of the underlying process. Supporting this, participants could not correct for the impact of username complexity when explicitly asked to do so. Three alternative explanations based on attributions of the variations in name complexity to seller origin (ingroup vs. outgroup), username generation method (seller personal choice vs. computer algorithm) and age of the eBay profiles (10 years vs. 1 year) were tested and ruled out. Finally, we show that manipulating the ease of reading product descriptions instead of the sellers’ names also impacts the trust ascribed to the sellers.

## Introduction

Can the mere name of a seller determine his trustworthiness in the eye of the consumer? Surely this is the case when the name is familiar and is reminiscent of prior interactions, or when its semantic meaning bears some emotional associations, such as a person named Johnny Goodfaith. But apart from that, can meaningless and superficial features of a seller’s name determine consumer attitudes? The present research explored this for the complexity of seller names. As an exemplary domain, we chose interactions in online marketplaces because in these contexts there are only few other pieces of information available than the name of a seller.

Commercial transactions frequently encompass some degree of uncertainty and consequently of risk for the buyer. For example, it is often the case that the seller has more information about the quality of the product or service he is providing than the buyer does. This information asymmetry opens the way for opportunistic behavior from a seller who might just take advantage of the other part (Akerlof, 1970; Mishra et al., 1998). Thus, as in other situations where risk is present, trust is a key aspect in buyer-seller relations, acting as a complexity reduction mechanism (e.g., Luhmann, 1979; Lewis and Weigert, 1985; Milne and Boza, 1999; Gefen, 2000) and enabling transactions when buyers cannot fully predict or control the intentions and actions of the sellers (e.g., Schurr and Ozanne, 1985; Yamagishi and Yamagishi, 1994; Mayer et al., 1995; Doney and Cannon, 1997; Gefen, 2000; Evans and Krueger, 2009, 2011; Thielmann and Hilbig, 2015).

When buyer-seller transactions occur in online marketplaces, uncertainty and risk might be even greater than in the traditional markets (Ridings et al., 2002; Grabner-Kräuter and Kaluscha, 2003; Beldad et al., 2010). This is due to the impersonal nature of online environments: usually, buyers and sellers never meet face-to-face, making it difficult to bind an online seller to a specific identity (Ridings et al., 2002; Beldad et al., 2010); and there is no information about the sellers’ standing in the community or the professionalism of the place from where they operate (online private sellers often run their business from their own home; Resnick et al., 2006). Also, there is not even the possibility to inspect the product personally and have a concrete idea of its real quality (Ba and Pavlou, 2002). This anonymous and impersonal context both increases the chances for dishonest and opportunistic behavior from the sellers’ side and makes the assessments of seller trustworthiness more difficult (Metzger et al., 2010). For these reasons, many online marketplaces like eBay or Amazon.com have put effort in developing systems that allow extracting the information that a seller will not take advantage of buyers. These reputation mechanisms gather the ratings or feedback scores of the online sellers’ previous interactions and transactions (for detailed descriptions of eBay’s reputation system, see Resnick et al., 2006; Cabral and Hortaçsu, 2010). There are already a significant number of studies investigating seller reputation showing that overall a good reputation increases not only the probability of sale but also final prices (see the summary of studies presented by Resnick et al., 2006).

To our knowledge, reputation seems to be the seller characteristic that has received most attention in the literature regarding trust building in online marketplaces (Ang et al., 2001; Shankar et al., 2002; Yoon, 2002; Corritore et al., 2003; Yousafzai et al., 2003; Cheema, 2008; Beldad et al., 2010; Metzger et al., 2010). Little is known about other features of the sellers that might affect perceptions about their trustworthiness and the likelihood that the buyers will trust and engage in transactions with them. But one indispensable and inevitable characteristic that buyers in online transactions have access to is the name of a seller. Based on psychological research on mental ease, which is outlined in the following section, we predicted that the mere complexity of a seller’s name will affect perceptions of his/her trustworthiness.

### Processing Fluency

Individuals’ attitudes are often influenced by the cognitive feelings triggered by the dynamics of their mental operations during information processing (Schwarz, 2004, 2015; Avnet et al., 2012). One particularly potent cognitive feeling is *processing fluency*, that is, the subjective feeling of mental ease that people experience while processing information (Reber et al., 2004; Alter and Oppenheimer, 2009). Mental ease can be elicited by different aspects of information, such as repetition, perceptual clarity, or writing font (for a review, see Alter and Oppenheimer, 2009). A high compared to low processing fluency triggers positive feelings (Garcia-Marques and Mackie, 2000), higher liking judgments (Reber et al., 1998), increases the truth-value ascribed to trivia statements (Reber and Schwarz, 1999; Silva et al., 2016, 2017) and elicits facial muscular activity specific to positive experiences (Harmon-Jones and Allen, 2001; Winkielman and Cacioppo, 2001; Lee and Labroo, 2004; Labroo et al., 2008; Topolinski et al., 2009). Thus, fluency seems to be inherently positive and that aura of positivity leads to more favorable evaluations of fluent as compared to disfluent information (the hedonic marking of fluency, Winkielman et al., 2003).

### Word and Name Pronounceability

Regarding the name of a seller, one key determinant of its fluency is its pronounceability. In psychological research, word pronounceability has been shown to affect consumer attitudes. For instance, Alter and Oppenheimer (2006) showed that the pronounceability of ticker codes of shares predicts short-term stock prices fluctuations both in the laboratory and with real-world stock market data, with stocks with easy-to-pronounce names outperforming stocks with difficult-to-pronounce names. Moreover, Song and Schwarz (2009) found that food additives are judged as more dangerous when they have difficult-to-pronounce rather than easy-to-pronounce names. Regarding person names more directly, Laham et al. (2012) showed that persons with easy-to-pronounce names are judged more positively and, according to a real world data analysis, they seem to occupy superior positions in companies’ hierarchies than persons with difficult-to-pronounce names. Also relevant to the present work, Newman et al. (2014) found that statements about different topics are given higher levels of truth when attributed to individuals with easy-to-pronounce names, and very recently Zürn and Topolinski (2017) showed that in economic trust games individuals trust more in partners bearing easy-to-pronounce names (for related effects of language articulation on attitudes, see Topolinski et al., 2014, 2015a,b; Topolinski and Boecker, 2016a,b).

Most recently Topolinski et al. (2016) have explored the impact of another, independent, contribution to processing fluency of nonsense words, namely the mere length of a word. They found that short anagrams were judged as more likely to be solved and as requiring less solvability effort than long anagrams. This demonstration, however, did not address attitudes but judgments of cognitive solvability of abstract intellectual problems.

### The Present Experiments

The present research explored whether mere complexity of seller names would influence perceived trustworthiness of those sellers. We chose the context of online marketplaces, such as the auction website eBay (Roth and Ockenfels, 2002; Ockenfels and Roth, 2006), because name complexity most likely plays an important role in this domain, due to the following. Usually, one searches in eBay for a certain product and the search generates a list of all available offers. When clicking through the offers, the obvious parameters of price and seller reputation vary across offers, but also the names of the sellers. Imagine you come across the seller SIBU and the seller VLEGTIQCLAPL. Both usernames are fairly meaningless and could simply be the result of a contraction of the sellers’ given and family names. However, given all other trustworthiness parameters being constant, would you expect one of them to be more trustworthy than the other?

Building on the evidence cited above, we predicted that: (1) Online seller profiles with easy- as compared to difficult-to-pronounce usernames will be evaluated as more trustworthy, and (2) Online seller profiles with short as compared to long usernames will be evaluated as more trustworthy. We tested these hypotheses in a series of experiments in which participants were presented with screenshots of ostensibly real profiles of eBay private sellers, rating them for trustworthiness.

Crucially, we explored the impact of username complexity in the face of more diagnostic information, with seller reputation being the most important one. Previous studies showed that private eBay sellers with an established good reputation receive a premium of up to 8.1 percent of the average selling price of their products, have a higher probability that individual bidders enter their auctions and also receive more bids (e.g., McDonald and Slawson, 2002; Bajari and Hortacsu, 2003; Resnick et al., 2006). More relevant to our study, buyers’ perceptions of eBay sellers’ trustworthiness are also positively affected by their reputation (Ba and Pavlou, 2002). Therefore, we manipulated the sellers’ reputation independently from name complexity and predicted that: (3) Online seller profiles with better as compared to worse reputation will be evaluated as more trustworthy.

Given the strong positive effect that seller reputation seems to have on seller success, it is possible that the effect of username complexity on trustworthiness evaluations is rather weak. Thus, finding evidence of such an effect will be quite remarkable. It suggests that the subjective experience of fluency associated with username complexity can influence buyers’ judgments of seller trustworthiness despite the presence of strong objective information attesting or disconfirming it. However, we hypothesize the effect of username complexity on consumers’ attitudes to be associated with the bias to evaluate fluently processed stimuli more positively (e.g., Winkielman et al., 2003; Lee and Labroo, 2004; Labroo et al., 2008), exerting its effects independent of the more systematic, conscious and deliberative use of objective arguments such as a seller’s prior reputation (Maheswaran et al., 1992; Bohner et al., 1994; Smith and DeCoster, 2000; Suri and Monroe, 2003; Strack and Deutsch, 2004; Strack et al., 2006; Novak and Hoffman, 2009). Moreover, these two different types of influences seem to relate to two different and independent types of trust, namely emotional trust (elicited by positive feelings about the target person, product or brand) and cognitive trust (elicited by rational arguments about the person, product or brand characteristics) (Lewis and Weigert, 1985). Thus, we also predicted that: (4) Name complexity effects will not be substantially qualified by seller reputation, in that seller reputation will not alter or reverse the predicted pattern of username pronounceability and length effects on trustworthiness (the two factors should have additive rather than interactive effects).

### Data Treatment and Power Analysis

We report all measures and conditions that were run in each Experiment. We report and justify exclusion of data (if any). We calculated required sample sizes *a priori* using G^∗^Power (Faul et al., 2007). To estimate the required sample size in a conservative fashion, we used the smallest effect of pronounceability found in Topolinski et al. (2016, Experiment 5) for the basic effect of pronounceability on anagram solvability difficulty, which was $\eta_{p}^{2}$ = 0.19. To replicate this effect with a power of 0.80, required sample size is *N* = 37. As the effect of pronounceability on perceptions of trustworthiness in consumer choices is unknown, we arbitrarily over-powered most of the present Experiments, with the smallest sample size being *N* = 38. Across all Experiments, analyses were run only after the full final sample size was collected.

### Ethics Statements and Open Data

Participation in all Experiments was voluntary. Participants gave their consent either when approached directly on the University campus or by clicking on the web link leading to the Experiments when invitations were disseminated online. Participants did not provide any identifying information and they could terminate their participation at any time. Experiments 8–10 were reviewed and approved by the Institutional Review Board at the University of Southern California (approval numbers: UP-15-00402 and UP-14-00630). Experiments 1–7 took place at the University of Cologne, Germany. These Experiments were reviewed and approved as part of the funding process by expert committees at the Department of Innovation, Science, and Research of the state of North Rhine-Westphalia (the German system does not require subsequent ethics review of individual studies and does not maintain institutional review boards for psychological experiments at the local university level).

All materials and databases are available online at https://osf.io/7mgaq.

## Experiment 1

In the first Experiment we orthogonally manipulated username pronounceability and seller reputation using simulated eBay profiles. We also explored the impact of seller repetition. It is often the case that individuals encounter the same sellers more than once when searching for a product in online auction sites. Repetition has been consistently shown to increase positive attitudes toward neutral and meaningless stimuli (the mere exposure effect, Zajonc, 1968; Monahan et al., 2000), other people (Rhodes et al., 2001; Zebrowitz et al., 2008) and also product brands (Janiszewski, 1993; Baker, 1999; Skurnik et al., 2005). Therefore, participants were requested to rate all the sellers twice. Based on the extensive previous evidence of repeated exposure effects, in addition to the predicted (1) pronounceability and (3) reputation effects, we predicted that: (5) Perceptions of trustworthiness will increase from the first to the second exposure to the eBay profiles.

### Method

#### Participants

Fifty-eight participants (32 men, *M_age_* = 24 years, *SD* = 1) were assigned to the conditions of a 3 (Pronounceability: easy- vs. medium- vs. difficult-to-pronounce usernames) × 2 (Reputation: good vs. bad) × 2 (Repetition: first evaluation vs. second evaluation of the profiles) factorial design, with all independent variables manipulated within-participants. Participants were volunteers recruited online by dissemination of the study in online platforms and mailing lists directed at university students in Germany.

#### Materials and Procedure

##### Usernames

Name stimuli were used as the eBay sellers’ usernames. A large pool of letter strings that formed meaningless words in the German language were construed so as to have a high to low vowel-consonant ratio and then arranging the letters into sequences that either did or did not conform to German phonotactic constraints (Brent and Cartwright, 1996; Saffran, 2003; Warker and Dell, 2006) according to the authors’ subjective judgment. For example, the word SIBU has two vowels and two consonants, thus having a high vowel-consonant ratio and a consonant is always followed by a vowel, making it fairly easy to read. In the word PTONBIA, the ratio is three vowels to four consonants, and two consonants appear in sequence before a vowel is added. Finally, the word VLEGTIQCLAPL has a ratio of only three vowels to nine consonants, and the letters are rearranged so that there is at least one sequence of three straight consonants. In an independent pilot study, these letter strings were pre-tested in how difficult they were to pronounce by an independent group of *N* = 13 judges (10 point scale, in which 1 = not difficult at all, and 10 = very difficult). The resulting ratings ranged from *M* = 1.00, *SD =* 0.00 and *M* = 9.92, *SD =* 0.28. We selected the 10 easiest (*M* = 1.30, *SD* = 0.77), 10 medium (*M* = 5.25, *SD* = 2.51) and the 10 most difficult (*M* = 9.44, *SD* = 1.00) to pronounce items to be used as usernames in the Experiment, making a total of 30 name stimuli (Supplementary Table 1 presents the complete list of usernames for Experiments 1 and 2).

To increase the credibility of our cover story that the profiles belonged to real eBay private sellers, random combinations of three digits were added to the usernames. This is a strategy often used by individuals when they register in a website and the username they chose is already in use (e.g., a user wanting to register as Ricot on eBay changes the username to Ricot083 when the original version is already taken by someone else).

##### Reputation

We manipulated seller reputation with a five star rating system (Thoma and Williams, 2013), which we believed would be very easy to understand by our participants given its wide use in several commercial domains (from hotels classification to reviews of both products and sellers in online marketplaces). The silhouette of five starts was presented in each seller profile. For good reputation sellers, either 5 or 4.5 of the star silhouettes were filled with a yellow-golden color; for bad reputation sellers only 3 or 3.5 star silhouettes were colored. The two different ratings in each reputation level were used to make the sellers slightly more diverse and were randomly assigned to the sellers. We opted for not having extremely bad reputation ratings of only 1 or 1.5 stars because feedback on real eBay users is most frequently quite positive and sellers get negative ratings only 1% of the time (Resnick et al., 2006).

Thirty eBay profiles were created using as a basis a screenshot of the standard seller profile image eBay presents next to every product that is auctioned (Supplementary Figure 1). The combination of the two independent variables resulted in six possible profiles: easy-pronunciation with good-reputation; easy-pronunciation with bad-reputation; medium-pronunciation with good-reputation; medium-pronunciation with bad-reputation; difficult-pronunciation with good-reputation; difficult-pronunciation with bad-reputation. There were five sellers for each type of profile, resulting in a total of 30 profiles. To assure that each of the easy-, medium- and difficult-to-pronounce usernames was rated for trustworthiness both with a good and a bad reputation score, two stimuli lists counter-balanced the assignment of the usernames to the two reputation conditions.

##### Procedure

Instructions informed participants that they were going to see several screenshots of private eBay sellers and that they should indicate how trustworthy they perceived each to be. Participants used a Likert-type rating scale presented below every eBay profile with the question “How trustworthy is this seller?” The scale ranged from 1 – not at all to 10 – very much. To guarantee that participants attended to the usernames and not only to their reputation (since it is likely that objective information about previous behavior has a great weight in trust related assessments, e.g., Ba and Pavlou, 2002; Resnick et al., 2006), participants were asked to carefully attend to all the information provided about the sellers, including the usernames as these could play a role later in the experiment^1^. In the first exposure, participants judged the 30 profiles in a random order for each participant. After this, without any break or further instruction, all 30 profiles were presented and judged again in a different new random order for each participant. After completing the evaluation, participants reported age and gender, and were asked to state what factors had influenced their evaluations.

### Results

**FIGURE 1:**
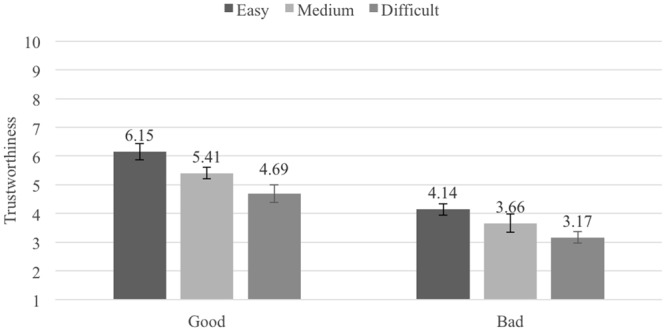
Average trustworthiness ratings in Experiment 1, by Pronounceability and Reputation conditions; error bars denote standard errors.

The mean trustworthiness ratings for the six Pronounceability × Reputation conditions are shown in **Figure 1** (across repetition levels). A 3 (Pronounceability: easy- vs. medium- vs. difficult-to-pronounce usernames) × 2 (Reputation: good vs. bad) × 2 (Repetition: first evaluation vs. second evaluation) repeated measures ANOVA (all factors within-participants) found the predicted main effects for Pronounceability, *F*(2,114) = 52.27, *p* < 0.001, $\eta_{p}^{2}$ = 0.48, and for Reputation, *F*(1,57) = 94.13, *p <* 0.001, $\eta_{p}^{2}$ = 0.62. There was also an interaction between the two factors, *F*(2,114) = 17.38, *p* < 0.001, $\eta_{p}^{2}$ = 0.23. The main effect of repetition was not statistically significant, *F*(1,57) = 1.50, *p* = 0.227, as well as none of the interactions involving this factor (all *F*s < 2, *p* > 0.300).

As **Figure 1** shows, eBay seller profiles with easy-to-pronounce usernames (*M* = 5.18, *SE* = 0.23) were considered more trustworthy than profiles with medium-difficult-to-pronounce usernames (*M* = 4.55, *SE* = 0.24), *t*(57) = 5.58, *p* < 0.001, *d_z_* = 0.73, 95% CI [0.40, 0.85], which in turn were considered more trustworthy than profiles with difficult-to-pronounce usernames (*M* = 3.97, *SE* = 0.26), *t*(57) = 7.71, *p* < 0.001, *d_z_* = 1.01, 95% CI [0.43, 0.74]. The main effect associated with reputation shows that across all pronounceability conditions profiles with good reputation (*M* = 5.44, *SE* = 0.30) were considered more trustworthy than profiles with bad reputation (*M* = 3.69, *SE* = 0.19). The pronounceability × reputation interaction shows that the impact of pronounceability was more extreme in the case of good reputation sellers. But the differences between easy- and medium-to-pronounce, as well as between medium- and difficult-to-pronounce usernames were highly significant in each of the reputation levels (all *t*s > 4.00, *p*s < 0.001).

Previous research shows that gender influences trusting behavior in economic contexts such as the Investment Game or the Trust Game, namely that men seem to trust more than women and women seem to be more trustworthy than men (Croson and Buchan, 1999; Buchan et al., 2008). Thus, we tested whether gender differences would emerge in our experimental paradigm. For each participant, we calculated a difference score between the trustworthiness ratings given to the easy-to-pronounce usernames and the difficult-to-pronounce usernames, collapsing across all other factors. We ran an independent samples *t*-test on this difference score, entering Participant gender (male vs. female) as the between-participants factor. Results showed that men and women did not differ in their trustworthiness evaluations, *t* < 1.^2^

### Discussion

These results clearly suggest that username pronounceability has a strong impact on how trustworthy an eBay seller may seem to potential buyers. The effect sizes we found were much larger than those in previous studies on pronounceability (e.g., Topolinski et al., 2016). Moreover, this effect emerged in a context with clear and unambiguous objective information about the sellers’ reputation. The absence of a repetition effect, however, was quite surprising in light of extensive work on the mere exposure effect (see Bornstein, 1989). But recent work has clarified that mere exposure effects are strong when repetition is varied within-participants, but weak or absent when repetition is varied between-participants (Dechêne et al., 2009, 2010; see also Hansen et al., 2008). This presumably reflects that the fluency experience varies from stimulus to stimulus in within-participants designs, where some stimuli are repeated and others are not. In contrast, repeating all or none of the stimuli reduces the variation in participants’ fluency experiences, thus making the experiential signal less informative. From this perspective, a repetition effect was not obtained in Experiment 1 because repetition did not vary from stimulus to stimulus. In contrast, pronounceability did and so it was able to elicit fluency effects. Experiment 2 was designed to address this issue and at the same time replicate the pronounceability effect.

## Experiment 2

Experiment 2 was a close replication of the previous Experiment, except that repetition’s effect was assessed by comparing trustworthiness ratings given to repeated vs. new seller profiles.

### Method

#### Participants

Fifty-eight participants (14 men, *M*_age_ = 22, *SD* = 3) were assigned to the conditions of a 3 (Pronounceability: easy vs. medium vs. difficult to pronounce usernames) × 2 (Reputation: good vs. bad) × 2 (Repetition: repeated vs. new profiles) factorial design, with all independent variables manipulated within-participants. Participants were student volunteers recruited on the university campus of a German university.

#### Materials and Procedure

To implement the changes in our design, 30 new eBay seller profiles were added to the previous ones. Thus, 30 new meaningless letter strings varying in pronunciation fluency (10 easy-, 10 medium- and 10 difficult-to-pronounce items, created with the same method described in Experiment 1) were added to the original pool of usernames. Four list of material were created so that repeated vs. new and good vs. bad status of the profiles were completely counterbalanced in the three levels of pronounceability. The procedure of Experiment 1 was fully replicated, except that on the second round of trustworthiness evaluations participants rated 30 new seller profiles mixed with the 30 old profiles.

### Results

**FIGURE 2:**
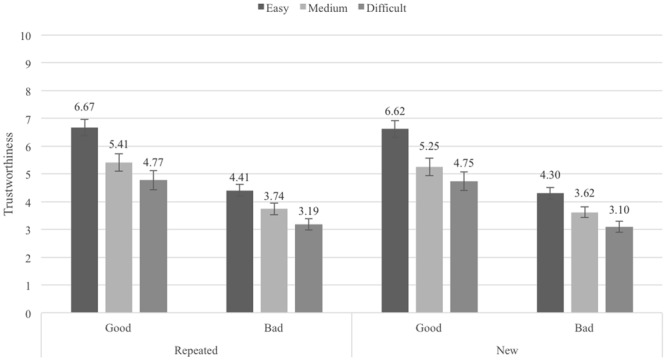
Average trustworthiness ratings in Experiment 2, by Pronounceability, Reputation and Repetition conditions; error bars denote standard errors.

We analyzed only the trustworthiness ratings of the second block of stimuli (with repeated and new profiles) because the effect of repetition could be assessed only in this block. The conditional means are shown in **Figure 2**. A 3 (Pronounceability: easy vs. medium vs. difficult to pronounce usernames) × 2 (Reputation: good vs. bad) × 2 (Repetition: repeated vs. new profiles) ANOVA (all factors within-participants) revealed again the main effect for Pronounceability, *F*(2,114) = 75.41, *p* < 0.001, $\eta_{p}^{2}$ = 0.57, the main effect for Reputation, *F*(1,57) = 88.25, *p <* 0.001, $\eta_{p}^{2}$ = 0.61, and the same interaction between the two factors, *F*(2,114) = 27.77, *p* < 0.001, $\eta_{p}^{2}$ = 0.33. Replicating Experiment 1, eBay sellers with easy-to-pronounce usernames (*M* = 5.50, *SE* = 0.23) were considered more trustworthy than sellers with medium-difficult-to-pronounce usernames (*M* = 4.51, *SE* = 0.24), *t*(57) = 9.01, *p* < 0.001, *d_z_* = 1.18, 95% CI [0.77, 1.21], which in turn were considered more trustworthy than sellers with difficult-to-pronounce usernames (*M* = 3.95, *SE* = 0.26), *t*(57) = 6.56, *p* < 0.001, *d_z_* = 0.86, 95% CI [0.39, 0.72]. Also as in Experiment 1, profiles with good reputation (*M* = 5.58, *SE* = 0.31) received higher ratings of trustworthiness than profiles with bad reputation (*M* = 3.73, *SE* = 0.19), across all pronounceability levels. The interaction between Pronounceability and Reputation factors also replicated the pattern found in Experiment 1, showing that the differences between the different levels of pronounceability were larger for good reputation sellers. Paired sample *t*-tests again demonstrated that the differences between easy- and medium-, as well as between medium- and difficult-to-pronounce usernames were highly significant in each of the two reputation levels and both for repeated and new profiles (all *t*s > 5.00, *p*s < 0.001). The main effect of repetition was now statistically significant, *F*(1,57) = 6.13, *p* = 0.016, $\eta_{p}^{2}$ = 0.10. Repeated profiles (*M* = 4.70, *SE* = 0.24) were considered more trustworthy than new profiles (*M* = 4.61, *SE* = 0.23). None of the interactions involving Repetition was significant (all *F*s < 1).

### Discussion

Results perfectly replicate Experiment 1 and show also an effect of repetition. Together, Experiments 1 and 2 suggest that fluency effects are more robust when fluency is manipulated within-participants and varies across stimuli (see Dechêne et al., 2009, 2010). Again, eBay profiles with easy-to-pronounce usernames were considered more trustworthy than profiles with difficult-to pronounce usernames, independently of their reputation and their familiarity. In Experiment 3 we explored the effect further, disentangling two independent parameters of name complexity, namely pronounceability and length.

## Experiment 3

In Experiments 1 and 2 participants judged eBay profiles with usernames that varied in pronunciation ease. But in those two Experiments these easy-, medium- and hard-to-pronounce letter strings had systematic variation in number of letters, or simply length: the easy-to-pronounce usernames were shorter (4 letters) than the medium-difficult-to-pronounce usernames (7 letters), which were in turn shorter that the difficult-to-pronounce usernames (12 letters). Given that most recent basic cognitive research studies showed that word pronounceability and word length have similar but independent effects in problem solving judgments (Topolinski et al., 2016), in Experiment 3 username pronounceability and length were manipulated orthogonally to disentangle their effects in perceptions of seller trustworthiness. Note that this manipulation is the first to disentangle the respective contributions of word pronounceability and word length on attitudes (Alter and Oppenheimer, 2006; Song and Schwarz, 2009; Laham et al., 2012; Newman et al., 2014). Thus, in this new Experiment, besides the replication of (1) pronounceability and (3) reputation effects, we also predicted (2) seller profiles with short usernames to be evaluated as more trustworthy than profiles with long usernames.

Given that the previous experiences revealed comparable significant differences on trustworthiness ratings between the three pronounceability levels, this Experiment focused solely on the comparison of the two extreme levels of that dimension (i.e., easy vs. difficult pronunciation).

### Method

#### Participants

Forty-eight participants (8 men, *M*_age_ = 23, *SD* = 4) were assigned to the conditions of a 2 (Pronounceability: easy- vs. difficult-to-pronounce usernames) × 2 (Length: short vs. long usernames) × 2 (Reputation: good vs. bad) factorial design, with all independent variables manipulated within-participants. Participants were student volunteers recruited on a German university campus.

#### Materials and Procedure

To manipulate username pronounceability and length independently, we used the 100 letter strings provided in Topolinski et al. (2016) for the experimental condition of unsolvable anagrams (i.e., non-anagrams; Supplementary Table 2). These letter strings were again meaningless and could not be re-arranged into real German words. Fifty of these letter strings were easy-to-pronounce and the other 50 were difficult-to-pronounce. In each pronounceability level, 25 items were short (6–8 letters) and 25 were long (9–11 letters) (for further details on how the material was created, see Topolinski et al., 2016). We again added random combinations of three digits to the usernames. The procedure was exactly like in Experiments 1 and 2, but the 25 usernames in each of the four Pronounceability × Length conditions were randomly associated with good vs. bad reputation profiles for each participant (eliminating the necessity to create multiple material lists counterbalancing the stimuli across conditions). The only other change was that the trustworthiness rating scale ranged from 1 to 9.

### Results

**FIGURE 3:**
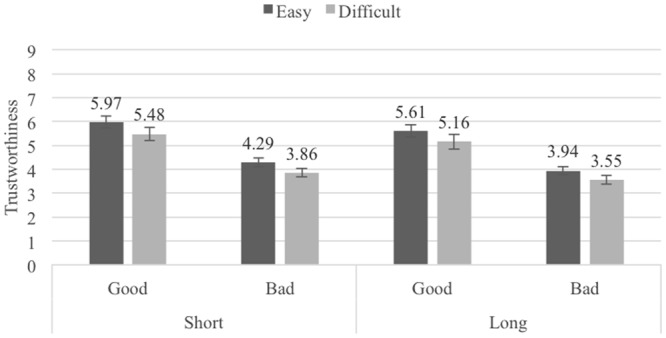
Average trustworthiness ratings in Experiment 3, by Pronounceability, Length and Reputation conditions; error bars denote standard errors.

The mean trustworthiness ratings for the Pronounceability × Length × Reputation conditions are shown in **Figure 3**. A 2 (Pronounceability: easy- vs. difficult-to-pronounce usernames) × 2 (Length: short vs. long) × 2 (Reputation: good vs. bad) repeated measures ANOVA (all factors within-participants) found a main effect for Pronounceability, *F*(1,47) = 18.16, *p* < 0.001, $\eta_{p}^{2}$ = 0.28, a main effect for Length, *F*(1,47) = 17.04, *p <* 0.001, $\eta_{p}^{2}$ = 0.27, and a main effect for Reputation, *F*(1,47) = 73.61, *p* < 0.001, $\eta_{p}^{2}$ = 0.61. Sellers with easy-to-pronounce usernames (*M* = 4.92, *SE* = 0.20) were again considered more trustworthy than sellers with difficult-to-pronounce usernames (*M* = 4.51, *SE* = 0.24), as were sellers with good reputation (*M* = 5.54, *SE* = 0.27) as compared to sellers with bad reputation (*M* = 3.89, *SE* = 0.16). The effect of Length was also as predicted, with short-username profiles (*M* = 4.88, *SE* = 0.18) being considered more trustworthy than long-username profiles (*M* = 4.55, *SE* = 0.21). None of the interactions between the three independent variables reached statistical significance (all *F*s < 2).

### Discussion

This Experiment shows that username pronounceability and length have strong, independent effects on the level of perceived trustworthiness attributed to online sellers. By disentangling the two dimensions and manipulating them orthogonally, the effect sizes were somewhat smaller than in the previous Experiments (except for the effect of Reputation), suggesting that indeed length might have contributed to the stronger effects previously observed for pronounceability. But note again that the two effects persisted despite the presence of the much more informative and seemingly powerful information of seller reputation.

After demonstrating the basic existence of the effects of username complexity (pronounceability and length) on trustworthiness, the remaining Experiments tested possible driving mechanisms.

## Experiment 4

The three previous Experiments show that username pronounceability and length are strong cues that individuals use to evaluate the trustworthiness of eBay sellers. In principle, these cues may exert their influence through a deliberate or intuitive pathway. On the one hand, participants may deliberately attend to the seller’s name and infer from high complexity that there is something odd about the seller. If so, explicitly informing participants that name complexity is unrelated to sellers’ trustworthiness should attenuate or eliminate the observed effects. On the other hand, name complexity may exert its influence through processing fluency, as we suggest. From this perspective, fluent processing elicits a positive response and is associated with a sense of familiarity, which foster increased trust. If so, telling people that name complexity (the external cue) is unrelated to sellers’ trustworthiness should have little effect if the judgment is based on the subjective experience it elicits (for a framework on how fluency and affect inform intuitive cognitive judgments, see Topolinski and Strack, 2009a,b,c).

### Method

#### Participants

Eighty-five participants (12 men, *M*_age_ = 23, *SD* = 5) were assigned to the conditions of a 2 (Pronounceability: easy- vs. difficult-to-pronounce usernames) × 2 (Length: short vs. long usernames) × 2 (Reputation: good vs. bad) × 3 (Correction instructions: pronounceability vs. length vs. no-correction; between-subjects) mixed factorial design. Participants were student volunteers recruited on a German university campus.

#### Materials and Procedure

Materials and procedure were identical to Experiment 3, except that different instructions were given to participants. Participants were randomly assigned to one of the following three conditions. In the *pronounceability-correction condition* (*n* = 27), participants were warned that the usernames varied in pronounceability, but that this factor has nothing to do with how trustworthy a seller is and so they should try to not let their judgments be affected by it. In the *length-correction condition* (*n* = 29), the same type of instructions was given for the length of the usernames. Finally, in the *no-correction condition* (*n* = 29) there was no particular instruction beyond the general information given in all the previous Experiments.

### Results

**FIGURE 4:**
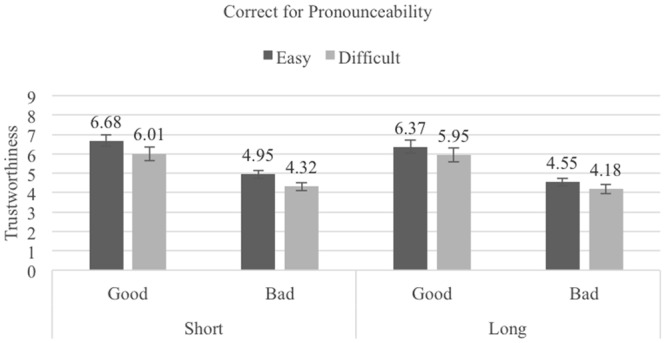
Average trustworthiness ratings in the Pronounceability-correction condition of Experiment 4; error bars denote standard errors.

**FIGURE 5:**
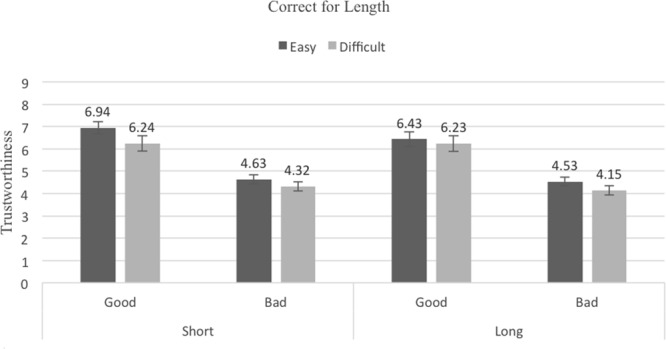
Average trustworthiness ratings in the Length-correction condition of Experiment 4; error bars denote standard errors.

**FIGURE 6:**
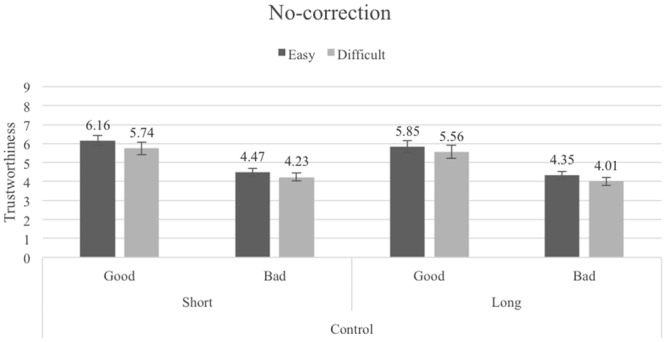
Average trustworthiness ratings in the No-correction condition of Experiment 4; error bars denote standard errors.

The mean trustworthiness ratings for the Pronounceability × Length × Reputation conditions are shown in **Figures 4–6**, (pronounceability-correction, length-correction and no-correction, respectively). A 2 (Pronounceability: easy- vs. difficult-to-pronounce usernames) × 2 (Length: short vs. long usernames) × 2 (Reputation: good vs. bad) × 3 (Correction instructions: pronounceability-correction vs. length-correction vs. no-correction) mixed ANOVA (last factor between-participants) revealed once more strong main effects associated with Pronounceability, *F*(1,82) = 34.17, *p <* 0.001, $\eta_{p}^{2}$ = 0.29, Length, *F*(1,82) = 31.41, *p <* 0.001, $\eta_{p}^{2}$ = 0.28, and Reputation, *F*(1,82) = 192.49, *p <* 0.001, $\eta_{p}^{2}$ = 0.70. These effects replicate what was found in the previous Experiments: participants attributed higher trustworthiness to sellers with easy- (*M* = 5.50, *SE* = 0.13) as compared to difficult-to-pronounce usernames (*M* = 5.05, *SE* = 0.15); to sellers with short- (*M* = 5.35, *SE* = 0.13) as compared to long-usernames (*M* = 4.15, *SE* = 0.14); and to sellers with a good (*M* = 6.14, *SE* = 0.18) as compared to bad (*M* = 4.36, *SE* = 0.11) reputation.

In addition, a significant interaction between Pronounceability and Length was found, *F*(1,82) = 5.70, *p =* 0.019, $\eta_{p}^{2}$ = 0.07. The pattern of means associated with this interaction shows only that although the difference between easy- and difficult-to-pronounce usernames is always significant, it is larger for short (*M_easy_* = 5.59, *SE* = 0.13, *M_difficult_* = 5.11, *SE* = 0.15, *t*(82) = 5.62, *p <* 0.000, *d_z_* = 0.61, 95% CI [1.86, 3.90]) than for long usernames (*M_easy_* = 5.31, *SE* = 0.14, *M_difficult_* = 4.98, *SE* = 0.15, *t*(82) = 4.96, *p <* 0.000, *d_z_* = 0.54, 95% CI [1.17, 2.74]). This interaction was further qualified, albeit only marginally, by Reputation, *F*(1,82) = 3.77, *p* = 0.056, $\eta_{p}^{2}$ = 0.04, in that only for sellers with good reputation was the Pronounceability × Length interaction significant [*F*(1,82) = 6.12, *p* = 0.015; interaction for bad reputation sellers, *F* < 1].

The independent effect of the Correction instruction was not significant (*F* < 1). Most importantly for this experiment, it also did not qualify the effects promoted by pronounceability, length and reputation (all *F*s < 2). As **Figures 4–6** show, the exact same pattern of effects was found in the three Correction instruction conditions. Instructing participants to not let their judgments be influenced by the pronounceability or length of the usernames did not eliminate the seemingly heuristic, irrational bias that these dimensions bear on trustworthiness evaluations.

### Discussion

This Experiment showed that username pronounceability and length influence evaluations of seller trustworthiness even when participants were explicitly asked to correct for them. These results suggest that name complexity effects on attitudes toward online sellers are not caused by a conscious and deliberative use of pronounceability or length as cues to infer trustworthiness. The route through which username complexity exerts its effects seems to be rather experiential (Novak and Hoffman, 2009), stemming from the positivity that is associated with fluent processing (e.g., Winkielman and Cacioppo, 2001; Winkielman et al., 2003; see also Topolinski and Strack, 2009a,b,c).

An alternative explanation is that participants maybe attributed the differences in username pronounceability and length to other characteristics of the sellers. If so, simply being warned that the usernames differed in how easy they were to pronounce or in their length could not eliminate the effects of other possible attributions participants might have made during their judgments. In the next three Experiments we addressed possible rational attribution processes, to further clarify the nature of name complexity influences on perceived trustworthiness.

## Experiment 5

In this Experiment we tested whether participants attributed name complexity to the origin of the sellers. Because the difficult-to-pronounce usernames do not conform to German phonotactic constraints and all the participants in previous Experiments were German students, it may have been that the more complex usernames were interpreted as belonging to sellers from foreign backgrounds with different language rules. Given that individuals hold more positive attitudes toward members of their ingroup than members of an outgroup (*ingroup bias*; Brewer, 1999; Everett et al., 2015), including higher expectations regarding ingroup members’ trustworthiness (see Balliet et al., 2014), it is possible that in the previous Experiments sellers with complex usernames were judged as less trustworthy because they were perceived as members of an outgroup and not because and not because their usernames were difficult-to-pronounce. This possibility finds support in studies using the economic Trust Game to explore trust and trustworthiness toward ingroup and outgroups members, which found evidence of ingroup bias in both measures (but only in Western countries like the United States of America; Buchan et al., 2006).

To test this hypothesis, we informed participants that some of the sellers were ostensibly located in Germany and some in Poland. Poles were chosen as the outgroup to the German participants because many negative stereotypes about this national group (such as “drunk and thieves”) are still present in the contemporary German society (Sakson, 2012). If the name complexity effects found so far are a result of ingroup–outgroup biases processes, then they should be eliminated or at least qualified by the information about the sellers’ origin.

### Method

#### Participants

Seventy-five participants (12 men, *M*_age_ = 23, *SD* = 4) were assigned to the conditions of a 2 (Pronounceability: easy- vs. difficult-to-pronounce usernames) × 2 (Length: short vs. long username) × 2 (Reputation: good vs. bad) × 2 (Seller origin: German vs. Poles) factorial design (all variables manipulated within-participants). Participants were student volunteers recruited on a German university campus.

#### Materials and Procedure

In this Experiment half of the eBay profiles were described as belonging to sellers from Germany and the other half to sellers from Poland. Thus, after the general instructions for the task, participants were told that the eBay sellers they were going to evaluate were German. Participants then evaluated half of the profiles (a random sample of 48 profiles across the Pronounceability × Length × Reputation conditions). After that a new instruction was given, saying that the next sellers were Polish. Sequence of the German vs. Polish block was randomly assigned to participants. Because we wanted to assure that the possible effects of ingroup–outgroup biases would not be corrected by participants wishing to answer in a more socially accepted manner, participants were instructed to evaluate the sellers spontaneously.

### Results

**FIGURE 7:**
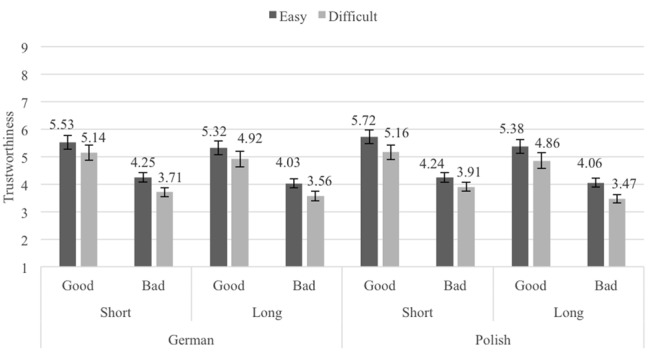
Average trustworthiness ratings in Experiment 5, by Seller Origin, Length, Pronounceability, and Reputation condition; error bars denote standard errors.

The mean trustworthiness ratings for the Pronounceability × Length × Reputation × Seller origin conditions are shown in **Figure 7**. A 2 (Pronounceability: easy- vs. difficult-to-pronounce usernames) × 2 (Length: short vs. long usernames) × 2 (Reputation: good vs. bad) × 2 (Seller origin: German vs. Poles) repeated measures ANOVA (all factors within-participants) was performed on the averaged trustworthiness ratings. The analysis revealed the same three main effects associated with Pronounceability, *F*(1,74) = 29.14, *p <* 0.001, $\eta_{p}^{2}$ = 0.28 (*M_easy_* = 4.82, *SE* = 0.18; *M_difficult_* = 4.34, *SE* = 0.20), Length, *F*(1,74) = 25.31, *p <* 0.001, $\eta_{p}^{2}$ = 0.26 (*M_short_* = 4.71, *SE* = 0.19; *M_long_* = 4.45, *SE* = 0.19), and Reputation, *F*(1,74) = 69.95, *p <* 0.001, $\eta_{p}^{2}$ = 0.49 (*M_good_* = 5.25, *SE* = 0.25; *M_bad_* = 3.91, *SE* = 0.14). The only significant effect associated with the Seller origin factor was a weak Length × Seller origin interaction, *F*(1,74) = 4.85, *p* = 0.031, $\eta_{p}^{2}$ = 0.06. However, this interaction only shows that the statistically significant difference between short and long usernames was larger when sellers were from Poland (*M_short_* = 4.76, *SE* = 0.19, *M_long_* = 4.44, *SE* = .19, *t*(74) = 5.20, *p <* 0.000, *d_z_* = 0.60, 95% CI [0.78, 1.75]) rather than from Germany (*M_short_* = 4.66, *SE* = 0.20, *M_long_* = 4.46, *SE* = 0.20, *t*(74) = 3.64, *p <* 0.000, *d_z_* = 0.42, 95% CI [0.36, 1.23]). No other effects reached significance (all *Fs* < 2.5, all *ps* > .125).^3^

### Discussion

This Experiment ruled out the hypothesis that the effects of name complexity on trustworthiness ratings are due to participants attributing complex usernames to foreigners or outgroup members (Laham et al., 2012). Seller origin did not qualify the effects of pronounceability or length in any conceptually relevant way. Thus, the ingroup bias hypothesis seems to be discarded as an alternative explanation. In Experiments 6 and 7, we explored two other possible seller attributes that participants might infer from our manipulations of username pronounceability and length.

## Experiment 6

In this Experiment we tested the hypothesis that participants rated sellers with complex usernames as less trustworthy because they infer that if sellers created usernames with seemingly random and non-linguistic combinations of letters, then this randomness or carelessness might transfer to the way they handle transactions. We tested this hypothesis by actively manipulating participants’ beliefs about username generation process. We informed participants either that the usernames presented in the profiles had been personally created and chosen by the sellers themselves, or that they had been automatically generated by an algorithm designed by eBay. If the effects we have observed in all the previous Experiments were in fact due to the inference that some sellers were so careless that they created such complex usernames, then pronounceability and length should impact trustworthiness ratings only in the condition where usernames were a personal choice of the sellers. When usernames are created through an automatic algorithm, it implies by necessity a lack of agency on the side of the sellers and consequently seller laziness or carelessness cannot play a role.

### Method

#### Participants

Forty participants (6 men, *M*_age_ = 22, SD = 3) were assigned to the conditions of a 2 (Pronounceability: easy- vs. difficult-to-pronounce usernames) × 2 (Length: short vs. long username) × 2 (Reputation: good vs. bad) × 2 (Belief about username generation method: personal choice vs. computer algorithm) factorial design (all variables manipulated within-participants). Participants were student volunteers recruited on the university campus to take part in a multiple experiments session in one of the university laboratories.

#### Materials and Procedure

Now participants were told that the profiles they were going to evaluate either had usernames the sellers had personally chosen and composed or usernames generated by eBay through an algorithm with no influence whatsoever from the sellers. Sequence of the Personal choice vs. Computer algorithm block was randomly attributed to participants. To assure participants kept in mind each of the two different instructions about username generation method, in each block they were asked to memorize how the usernames had been created. A manipulation check was introduced at the end of each block, asking participants to write the username generation method of the sellers they had just evaluated.

### Results

#### Manipulation Check

Only 52.5% (*n* = 21) of the participants correctly reported different username generation methods for the two blocks. Probably due to a misunderstanding of the question at the end of each block (“How were the usernames in this block construed?”), many participants did not report the exact information that had been given. An inspection of participants’ responses shows that they focused on the objective characteristics of the usernames, and thus most often replied “random letters and three digits.” To address this low rate of the manipulation check and avoid the noise it could bring to our data, we coded the participants in two groups according to whether they responded correctly to the manipulation checks (*n* = 21) or not (*n* = 19). We first analyzed the data including this variable as a between-participants factor to check for its potential effects. However, there was neither a main effect nor any significant interactions associated with this factor, and thus we removed it from the main statistical analysis.

#### Main Results

**FIGURE 8:**
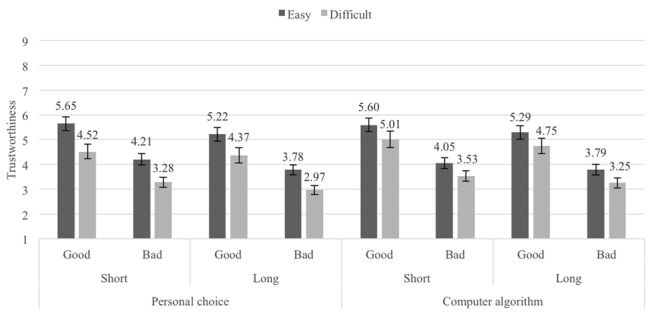
Average trustworthiness ratings in Experiment 6, by Belief in username generation method, Length, Pronounceability and Reputation conditions; error bars denote standard errors.

The mean trustworthiness ratings for the Pronounceability × Length × Reputation × Username generation method conditions are shown in **Figure 8**. A 2 (Pronounceability: easy- vs. difficult-to-pronounce usernames) × 2 (Length: short vs. long usernames) × 2 (Reputation: good vs. bad) × 2 (Belief about username generation method: personal choice vs. computer algorithm) repeated measures ANOVA (all factors within-participants showed the same strong three main effects associated with username Pronounceability, *F*(1,39) = 22.92, *p <* 0.001, $\eta_{p}^{2}$ = 0.37 (*M_easy_* = 4.70, *SE* = 0.20; *M_difficult_* = 3.96, *SE* = 0.22), Length, *F*(1,39) = 21.52, *p <* 0.001, $\eta_{p}^{2}$ = 0.36 (*M_short_* = 4.48, *SE* = 0.20; *M_long_* = 4.18, *SE* = 0.20), and Reputation, *F*(1,39) = 60.35, *p <* 0.001, $\eta_{p}^{2}$ = 0.61 (*M_good_* = 5.05, *SE* = 0.26; *M_bad_* = 3.61, *SE* = 0.17). The only significant effect associated with the Belief about username generation method was an interaction between this factor and Pronounceability, *F*(1,39) = 14.80, *p <* 0.001, $\eta_{p}^{2}$ = 0.28. This interaction only shows that the significant difference between easy- and difficult-to-pronounce usernames was larger when participants were told that the usernames had been personally chosen by the sellers (*M_easy_* = 4.71, *SE* = 0.22, *M_difficult_* = 3.78, *SE* = 0.22, *t*(39) = 5.17, *p <* 0.001, *d_z_* = 0.82, 95% CI [2.36, 5.08]) rather than randomly generated by an algorithm (*M_easy_* = 4.68, *SE* = 0.21, *M_difficult_* = 4.14, *SE* = 0.24, *t*(39) = 3.52, *p <* 0.001, *d_z_* = 0.56, 95% CI [0.93, 3.45]). No other effects reached significance (all *Fs* < 2, all *ps* > 0.20).^4^

### Discussion

As in Experiment 5, the alternative explanation that username complexity affected trustworthiness ratings due to some strategic attribution was shown to be unlikely. We observed reliable and strong significant effects for both name pronounceability and name length independent of whether participants thought the usernames had been created by the sellers themselves or by an automatic algorithm. It is true that the pronounceability effects was even stronger when participants thought the sellers had created the usernames themselves, but it was still significant when participants thought the usernames had been created by an algorithm. Also, the other determinant of name complexity, length, did not interact with belief of username generation method. So, once more, a mechanistic assumption is bolstered in that the fluency experience caused by pronounceability and length seems to directly influence trustworthiness ratings in an experiential manner (Novak and Hoffman, 2009).

## Experiment 7

One other possible rational inference participants may render from username complexity is to attribute it to the experience of the seller on eBay, with the following logic. As more and more sellers register with a website, the availability of usernames decreases and new users need to find more diverse alternatives for a name. This often leads to the need of creating distinct and extreme names that necessarily depart more and more from linguistic norms. Thus, participants might have inferred that seller profiles with difficult-to-pronounce and long usernames had been created more recently than easy-to-pronounce and short usernames. This in turn might have led to the assumption that sellers with more recently created accounts would be less experienced and thus less trustworthy (e.g., it is assumed that more experienced sellers have better quality products and are also better in describing their products accurately; Resnick et al., 2006).

To address this hypothesis, we manipulated the belief participants held about the year in which the eBay profiles were registered. If the level of experience that the sellers have was inferred from our name complexity manipulations, then the effects we observed previously should be at least qualified by that factor.

### Method

#### Participants

Thirty-eight participants (8 men, *M*_age_ = 22, *SD* = 3) were assigned to the conditions of a 2 (Pronounceability: easy- vs. difficult-to-pronounce usernames) × 2 (Length: short vs. long username) × 2 (Reputation: good vs. bad) × 2 (Age of eBay profile: 10 years vs. 1 year) factorial design (all variables manipulated within-participants). Participants were student volunteers recruited on the university campus to take part in a multiple experiments session in one of the university laboratories. Due to an error of codification, two participants were recorded with the same ID number and had to be deleted. Therefore, the final sample size was *N* = 36.

#### Materials and Procedure

Materials and procedure replicated Experiment 6, except that now the eBay profiles were presented either as having been registered in the year 2005 (older, well-established accounts) or in the year 2015 (accounts created close to one year before at the date of data collection). Sequence of Age of the eBay profiles blocks was randomly attributed to participants.

### Results

#### Manipulation Check

Seven participants did not enter the correct responses for the Age of eBay profiles manipulation checks and were thus discarded. Final sample size considered for the main analysis is *N* = 29.

#### Main Results

**FIGURE 9:**
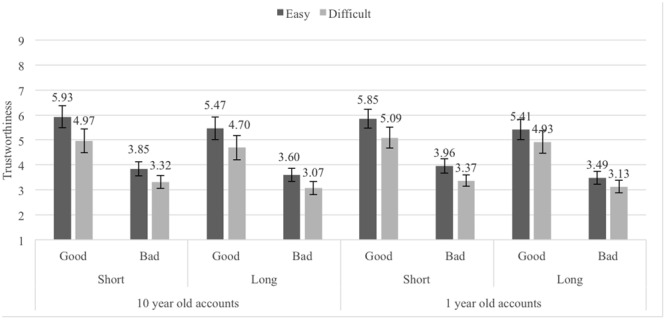
Average trustworthiness ratings in Experiment 7, by Age of the eBay accounts, Length, Pronounceability and Reputation conditions; error bars denote standard errors.

The mean trustworthiness ratings for the Pronounceability × Length × Reputation × Age of eBay profile conditions are shown in **Figure 9**. A 2 (Pronounceability: easy- vs. difficult-to-pronounce usernames) × 2 (Length: short vs. long usernames) × 2 (Reputation: good vs. bad) × 2 (Age of eBay profile: 10 years vs. 1 year) repeated measures ANOVA (all factors within-participants) again revealed the same main effects associated with username Pronounceability, *F*(1,28) = 18.95, *p <* 0.001, $\eta_{p}^{2}$ = 0.40 (*M_easy_* = 4.70, *SE* = 0.31; *M_difficult_* = 4.07, *SE* = 0.33), Length, *F*(1,28) = 17.44, *p <* 0.001, $\eta_{p}^{2}$ = 0.38 (*M_short_* = 4.54, *SE* = .30; *M_long_* = 4.23, *SE* = 0.32), and Reputation, *F*(1,28) = 43.59, *p <* 0.001, $\eta_{p}^{2}$ = 0.61 (*M_good_* = 5.29, *SE* = 0.42; *M_bad_* = 3.48, *SE* = 0.23). The interaction between Pronounceability × Reputation was also significant, *F*(1,28) = 13.88, *p <* 0.001, $\eta_{p}^{2}$ = 0.33, showing only that the significant difference between easy- and difficult-to-pronounce usernames was larger for good (*M_easy_* = 5.67, *SE* = 0.40, *M_difficult_* = 4.92, *SE* = 0.45, *t*(28) = 4.95, *p <* 0.001, *d_z_* = 0.92, 95% CI [1.74, 4.23]) than for bad reputation sellers (*M_easy_* = 3.73, *SE* = 0.26, *M_difficult_* = 3.22, *SE* = 0.23, *t*(39) = 3.50, *p <* 0.001, *d_z_* = 0.65, 95% CI [0.83, 3.18]). No other effects reached significance (all *Fs* < 2.5, all *ps* > .110).^5^

### Discussion

We found no moderation of the effects of name complexity by the Age of accounts factor. This suggests that the biasing effects of username pronounceability and length did not affect trustworthiness via attributions of less experience to sellers with more complex usernames.

## Experiments 8 and 9

In Experiments 8 and 9, we examined the generalizability of the username complexity effect. We used stimuli sampled from the real world and a different measure of seller trustworthiness. In Experiment 8, we asked people to evaluate real eBay seller usernames to examine whether the effect held for real eBay accounts. In Experiment 9, we asked people to evaluate real names rather than seller usernames. In both Experiments, we also examined whether the name complexity effect would hold when people evaluated how concerned they were about purchasing from these sellers, rather than directly asking them about trustworthiness. Since in Experiments 3–7 pronounceability and length of the seller usernames promoted independent and comparable effects, in the next Experiments we focused solely on the pronounceability component of name complexity.

### Experiment 8

#### Method

##### Participants

Fifty-four participants (35 men, *M*_age_ = 34, *SD* = 9) completed the study and rated names on two dimensions (concern about purchasing, and ease of pronunciation). Participants were recruited through Amazon’s Mechanical Turk.

##### Materials and procedure

We created a list of real eBay seller usernames by searching for digital cameras on eBay. We selected the first 40 usernames from the category of cameras priced at between $199 and $500 (Supplementary Table 3). Participants were told that they were going to see a series of e-commerce seller names from eBay. They were asked to imagine that they were trading with each seller. Then, participants completed two rating tasks. First, we asked them to estimate how concerned they would be about making a purchase from each of the sellers on a 7 point scale (1 = not concerned at all, 7 = very concerned). Second, we asked participants to rate how easy it was to pronounce each name, using a 7 point scale (1 = very difficult, 7 = very easy).

#### Results

We first examined whether participants’ ratings of the pronounceability of a seller’s username were associated with participants’ concern about buying from the seller. We used an item analysis approach and correlated the mean rating of pronunciation ease with the mean rating of concern of buying for each seller username. As expected, we found that the more difficult it was to pronounce a name, the more concerned people were about purchasing from that seller, *r*(38) = -0.85, *p* < 0.001.

#### Discussion

These results replicate the findings in Experiments 1–7 and extend those findings to real eBay usernames. The finding that the seller username pronounceability influenced people’s concern in interacting with the seller suggests that the previously observed effect of name complexity and disfluency on trustworthiness extends to more general concerns about the seller.

### Experiment 9

#### Method

##### Participants

Fifty-three participants (36 men, *M*_age_ = 32, *SD* = 10) were assigned to the conditions of a 2 (Pronounceability: easy- vs. difficult-to-pronounce usernames) within-participants design. Participants were recruited through Amazon’s Mechanical Turk.

##### Materials and procedure

We created a database of names by searching online newspapers from all over the world. From this database, we selected easy- and difficult-to-pronounce names from different regions of the world, until we had a set of 23 pairs of names with a similar number of letters and syllables. One constraint in selecting the names was that there was always an easy and difficult name from each region. We normed these names with 44 Mechanical Turk workers. Subjects saw 46 names in total and rated how easy it was to pronounce each name on a scale from 1 (easy) to 5 (difficult). The order of names was randomized for each subject. Using an item analysis we found that the difficult names (*M* = 3.01, *SE* = 0.10) were significantly more difficult to pronounce than the easy names (*M* = 2.06, *SE* = 0.09), *t*(44) = 7.26, *p* < 0.001, *d_z_* = 1.08, 95% CI [0.71, 1.45]. We use these pronunciation categories (easy- vs. difficult-to-pronounce names) to group names for analysis in Experiment 9 (Supplementary Table 4).

We told participants that “In a moment, we will show you a series of names from around the world. Each of these names is a seller that you can buy from on eBay or Etsy. Your task today is to imagine you are going to make a purchase from these sellers. For each seller name, we would like you to rate how concerned or cautious you would be about making a purchase from that person.” Participants saw 46 names, half easy- and half difficult-to-pronounce, in a random order. Participants were asked to rate on a 5-point scale, how concerned they would be about buying a product from each seller (1 = not concerned to 5 = very concerned).

#### Results

Replicating Experiments 1–8, we found that participants were less concerned to buy from sellers with easy-to-pronounce names (*M* = 2.68, *SE* = 0.12) than sellers with difficult-to-pronounce names (*M* = 2.83, *SE* = 0.12), *t*(52) = 3.57, *p* = 0.001, *d_z_* = 0.49, 95% CI [0.20, 0.77].

We also examined the influence of the region from which the names originated. It is possible that region somehow interacted with the ease of pronunciation to eliminate effects of name pronounceability for certain countries. A 2 (Pronounceability: easy- vs. difficult-to-pronounce usernames) × 7 (Region: Eastern Asia, Southern Asia, Eastern Europe, Western Europe, Middle East, Southeastern Africa, Northern Africa) repeated measures ANOVA (all factors within-participants) revealed a main effect of Pronounceability, *F*(1,32) = 8.78, *p* = 0.01, $\eta_{p}^{2}$ = 0.22 (*M_easy-to-pronounce_* = 2.68, *SE* = 0.04; *M_difficult-to-pronounce_* = 2.83, *SE* = 0.04), and Region, *F*(6,32) = 5.66, *p* < 0.001, $\eta_{p}^{2}$ = 0.52. The Pronounceability x Region interaction was not significant, *F*(6,32) = 1.24, *p* = 0.31, $\eta_{p}^{2}$ = 0.19. The main effect of Region shows that people were most concerned about buying from sellers with Middle Eastern names (*M* = 2.90, *SE* = 0.07) and least concerned about buying from sellers with Western European names (*M* = 2.47, *SE* = 0.06). But the absence of an interaction indicates that the effect of pronunciation held within each region, further illustrating its robustness.

#### Discussion

These findings demonstrate that the effect of pronunciation on trust is not unique to seller usernames, and suggest that when the seller’s actual name is available, it may influence people in the same direction. These findings also serve as a conceptual replication of Experiment 5 where the effect of pronunciation ease held, regardless of the perceived—or in Experiment 9—real origin of the names.

## Experiment 10

So far, all fluency manipulations pertained to the name of the seller. In the final Experiment we examined whether the ease of processing product information, rather than seller information, may also influence trust and willingness to buy from the seller. If the disfluency of product information is experienced as a problem signal, it may not only hurt the evaluation of the product but also consumers’ trust in the seller who offers it.

### Method

#### Participants

Ninety-nine participants (25 men, *M*_age_ = 20, *SD* = 2) were assigned to the condition of a 2 (Seller: B vs. C; within-participants) × 2 (Processing ease condition: Seller B easy-to-read/Seller C difficult-to-read vs. Seller B difficult-to-read/Seller C easy-to-read; between-participants) factorial design. Participants were student volunteers recruited on the University of Southern California campus to take part in a multiple experiments session in one of the university laboratories.

#### Materials and Procedure

We created three mockup eBay product offers. The first offer (Seller A) always served as a filler; it described a tripod and was easy to read. The second (Seller B) and third (Seller C) offers described compact digital cameras and were our critical items. One of these descriptions was presented in an easy-to-read style, and one was presented in a difficult-to-read style (e.g., italicized font, low contrast between text and background; see Supplementary Figures 2, 3). We counterbalanced so that each of the critical descriptions (Sellers B and C) appeared equally often in each processing ease condition.

Each offer appeared as a full-screen webpage. Participants were asked to study each offer as if they were actually thinking about buying one of the products. After studying these webpages, participants were asked to make several ratings about the camera offers. While making these ratings on 7-point scales participants saw a small thumbnail picture of each webpage as a reminder. The ratings pertained to: (1) how much they trusted the seller (1 = a little, 7 = a lot); (2) provided they would like to buy a camera, would they buy it from this seller (1 = not very likely, 7 = Very likely); (3) the reliability of the information presented on the website (1 = not reliable at all, 7 = very reliable); (4) how safe their credit card information would be at this website (1 = not safe at all, 7 = very safe); (5) how much they liked the webpage they had seen (1 = dislike 7 = like a lot), (6); how attractive each camera was, (1 = not attractive, 7 = very attractive), and (7) how easy it was to process information on the product page (1 = difficult, 7 = easy).

### Results

The ratings given to the two target product descriptions were analyzed with a mixed design repeated-measures ANOVA with Seller (B vs. C) as a within-participants factor and Processing ease condition (Seller B easy-to-read/Seller C difficult-to-read vs. Seller B difficult-to-read/Seller C easy-to-read) as a between-participants factor. Mean ratings for the fluent and disfluent seller, the interaction terms of the ANOVA and the follow-up simple *t*-tests are presented in **Table 1**. Confirming the effectiveness of the fluency manipulation, results showed that participants rated the offer in the easy-to-read style as easier to process than the offer in the difficult-to-read style (see bottom row of **Table 1**).

**Table 1:** Mean ratings for the fluent and disfluent sellers in Experiment 10, interaction terms of the ANOVA and follow-up analyses.

DVs		Seller				Analyses	Analyses
		B		C		Interaction term	Follow-up
		*M*	*SE*	*M*	*SE*
Trust in seller	Fluent	5.33	0.160	5.31	0.170	*F*(1,97) = 60.18, *p* < 0.001; $\eta_{p}^{2}$ = 0.38	B_fluent_ > C_Disfluent:_ *t*(47) = 6.19, *p* < 0.001, *d_z_* = 0.90, 95% CI [0.55, 1.23]
	Disfluent	4.49	0.200	3.73	0.230		C_fluent_ > B_Disfluent:_ *t*(50) = 4.55, *p* < 0.001, *d_z_* = 0.64, 95% CI [0.33, 0.94]
Willingness-to-buy	Fluent	5.06	0.220	4.98	0.200	*F*(1,97) = 40.58, *p* < 0.001, $\eta_{p}^{2}$ = 0.30	B_fluent_ > C_Disfluent_ : *t*(47) = 5.01, *p* < 0.001, *d_z_* = 0.72, 95% CI [0.40, 1.04]
	Disfluent	4.02	0.240	3.44	0.220		C_fluent_ > B_Disfluent_ : *t*(50) = 3.86, *p* < .001, *d_z_* = 0.54, 95% CI [0.24, 0.83]
Reliability of information	Fluent	5.42	0.964	5.25	1.146	*F*(1,97) = 34.51, *p* < 0.001, $\eta_{p}^{2}$ = 0.26	B_fluent_ > C_Disfluent_ : *t*(47) = 4.92, *p* < 0.001, *d_z_* = 0.71, 95% CI [0.39, 1.02]
	Disfluent	4.73	1.429	3.96	1.584		C_fluent_ > B_Disfluent_ : *t*(50) = 3.05, *p* < 0.001, *d_z_* = 0.43, 95% CI [0.14, 0.71]
Credit card information safety	Fluent	5.08	1.285	4.80	1.265	*F*(1,97) = 22.32, *p* < 0.001, $\eta_{p}^{2}$ = 0.19	B_fluent_ > C_Disfluent_ : *t*(47) = 4.44, *p* < 0.001, *d_z_* = 0.64, 95% CI [0.33, 0.95]
	Disfluent	4.55	1.527	4.02	1.828		C_fluent_ > B_Disfluent_: *t*(50) = 1.69, *p* = 0.10, *d_z_* = 0.24, 95% CI [-0.04, 0.51]
Website liking	Fluent	4.94	1.040	5.02	1.122	*F*(1,97) = 80.12, *p* < 0.001, $\eta_{p}^{2}$ = 0.45	B_fluent_ > C_Disfluent_ : *t*(47) = 6.32, *p* < 0.001, *d_z_* = 0.91, 95% CI [0.57, 1.25]
	Disfluent	3.37	1.612	3.29	1.637		C_fluent_ > B_Disfluent_: *t*(50) = 6.35, *p* < 0.001, *d_z_* = 0.89, 95% CI [0.56, 1.25]
Cameras attractiveness	Fluent	5.10	1.225	4.61	1.429	*F*(1,97) = 36.47, *p* < 0.001, $\eta_{p}^{2}$ = 0.27	B_fluent_ > C_Disfluent_ : *t*(47) = 4.18, *p* < .001, *d_z_* = 0.60, 95% CI [0.29, 0.91]
	Disfluent	3.71	1.628	4.06	1.420		C_fluent_ > B_Disfluent_ : *t*(50) = 4.37, *p* < 0.001, *d_z_* = 0.61, 95% CI [0.31, 0.91]
Ease of processing	Fluent	4.89	0.230	5.65	0.160	*F*(1,97) = 40.22, *p* < 0.001, $\eta_{p}^{2}$ = 0.29	B_fluent_ > C_Disfluent_ : *t*(47) = 2.55, *p* = 0.01, *d_z_* = 0.37, 95% CI [0.07, 0.66]
	Disfluent	3.86	0.280	4.31	0.230		C_fluent_ > B_Disfluent_ : *t*(50) = 6.12, *p* < 0.001, *d_z_* = 0.86, 95% CI [0.53, 1.18]

**Table 1** further shows that the seller with the easy-to-read product description had an advantage over the seller with the difficult-to-read description on each of the dependent variables. Most importantly, participants trusted seller B more than seller C when offer B was easier to read, but trusted seller C more than B when offer C was easier to read. Going beyond the measures used in the earlier Experiments, participants also reported a higher likelihood to purchase from the seller with the easier to read offer, considered the product information more reliable, and their credit card information in safer hands. They also liked the camera, as well as the webpage, more when the product offer was easy to read, consistent with earlier findings on the beneficial effects of processing fluency on esthetic appreciation (Reber et al., 2004).

### Discussion

Whereas the preceding Experiments manipulated ease of processing by varying the ease with which a seller’s username could be pronounced, Experiment 10 varied the ease of reading product descriptions. It found beneficial effects of processing fluency across a range of dependent variables. Consistent with the preceding Experiments, when the product description was easy to read, participants reported more trust in the seller, a higher likelihood of buying from the seller and fewer concerns about the safety of their credit card information than when the same product description was difficult to read. To our knowledge, this is the first study to document such pervasive effects of the ease of processing product information on the perceived trustworthiness of the seller. As observed in other domains (for a review, see Schwarz, 2015), people are likely to bring their metacognitive experiences to bear on any judgment to which they are applicable, giving the print font of a product description the potential to affect the trustworthiness of the seller (as observed in this Experiment) and the ease of pronouncing the seller’s username the potential to affect judgments of the product.

## General Discussion

We tested the impact of username complexity of eBay seller profiles on trustworthiness perceptions, using different measures of trust in market place interactions. Across nine Experiments we found that eBay profiles with less complex usernames were consistently rated as more trustworthy than profiles with more complex usernames. This was even observed when diagnostic information about the good or bad reputation of the sellers was available (Experiments 1–7). Two of our Experiments established external validity of the stimuli by sampling real usernames from eBay (Experiment 8) and real person names from different world regions (Experiment 9). Experiments 8–9 generalized the findings to older participants (*M*_age_ of 34 and 32 years) than those in Experiments 1–7 and 10 (*M*_age_ between 20 and 24 years), which is in line with findings in economic games showing that trust and trustworthiness remain constant across adulthood (Sutter and Kocher, 2007). Going beyond the ease of processing a seller’s name, we further found that difficulty in processing a product description can also adversely affect perceptions of a seller’s trustworthiness (Experiment 10).

Our name complexity studies are the first to systematically disentangle two aspects of name complexity, namely the contributions of name pronounceability and name length (Song and Schwarz, 2009; Laham et al., 2012; Newman et al., 2014). They show that both components exert independent effects, with easy-to-pronounce and short names being rated as more trustworthy than hard-to-pronounce and long names. We further tested whether participants had insight into the name complexity effect and could correct for it when explicitly warned that they should not let their evaluations be influenced by the seller’s name (Experiment 4). This was not the case. From the perspective of mental correction models (Strack and Hannover, 1996; Wegener and Petty, 1997; for a review, see Schwarz, 2015) this suggests that people lack a lay theory that entails that difficult names impair trust and hence cannot systematically correct for the influence of this variable when made aware of it. Note that such warnings are different from misattribution manipulations, which explicitly offer an alternative source for experienced difficulty, thus undermining the informational value of the experience (e.g., Novemsky et al., 2007). Hence, misattribution manipulations can elicit discounting effects even when people are unaware that the experiential input may influence their judgment – they simply change the diagnosticity of the experiential input. In contrast, warnings merely draw attention to a potential input and any correction requires insight into the direction and size of its likely impact. Accordingly, the influence of ease of processing variables can be more easily eliminated through misattribution manipulations (e.g., Schwarz et al., 1991; Sanna et al., 2002; Novemsky et al., 2007) than corrected in response to warnings (Experiment 4). A misattribution of affect manipulation could also help to clarify the role that the experience of positive affect that is associated with fluent processing (Winkielman and Cacioppo, 2001) plays on the effects of our Experiments.

Our studies further indicate that the name complexity effect cannot be traced to inferences about objective features of the seller that may bear on the seller’s perceived trustworthiness, including origin and carefulness of the seller and age of the online account; neither of these variables moderated the name complexity effect (Experiments 5–7). This observation is consistent with the conceptual rationale of fluency based judgment: fluently processed material appears more familiar than disfluently processed material (e.g., Song and Schwarz, 2009), elicits a more positive affective response (e.g., Winkielman and Cacioppo, 2001), and is associated with less attention to details of the message (e.g., Song and Schwarz, 2008) and higher acceptance of the message as true (e.g., Reber and Schwarz, 1999; McGlone and Tofighbakhsh, 2000; Silva et al., 2016; for a review, see Schwarz, 2015). As observed in Experiment 10, seller trustworthiness also benefits from fluent processing even when the processed information is about the product rather than the seller him- or herself.

To our knowledge, this set of studies is the first demonstration of how other seller characteristics beyond the well-researched objective reputation information (Ba and Pavlou, 2002; Corritore et al., 2003; Resnick et al., 2006; Cheema, 2008) can determine trustworthiness in online marketplaces. As such, our findings are an important addition to other research dealing with trust building in online commercial contexts (e.g., Ang et al., 2001; Shankar et al., 2002; Yoon, 2002; Corritore et al., 2003; Grabner-Kräuter and Kaluscha, 2003; Yousafzai et al., 2003; Beldad et al., 2010; Metzger et al., 2010). Our Experiments provide the first evidence that subjective experiences of mental ease associated with the mere reading of easy-to-pronounce seller (user)names and of product descriptions increases perceptions of online trustworthiness, and can thus ultimately increase the likelihood that a seller is chosen as a transaction partner (trustworthiness signals approach behavior; see Todorov, 2008).

### Implications of the Present Findings

Our findings have important practical implications in many consumer-related contexts. The most obvious is of course connected with name composition and choice. In many everyday situations, the first information that is available about others is their name, be it in form of an email, when the employee of a customer care service identifies himself, or in the nametags worn during professional meetings. Globally, our results suggest that individuals should strive for simple, easy-to-pronounce names as a way to increase perceived trustworthiness, which in turn may contribute to positive outcomes. Similar to Laham et al. (2012) findings that individuals with easy-to-pronounce names are liked more and attain higher positions in their companies’ hierarchy, it is possible that consumers are more persuaded to try a new product in their local store when approached by a saleswoman with an easy-to-pronounce name depicted in her nametag.

The importance of choosing a fairly simple, short and easy-to-pronounce name might be even greater in e-commerce and online markets. Because in these contexts there are even fewer pieces of information both about transaction partners and the quality of the products (Degeratu et al., 2000), an easy-to-pronounce username might just boost perceptions of trustworthiness enough to enable more opportunities for successful transactions. An easy-to-pronounce name might thus work as a success-enabling tool. One way in which these effects could be assessed is by gathering real data from auction sites like eBay or Amazon.com to analyze the correlation between sale success, price premiums, and username pronounceability (as often done in research on seller reputation; Cabral and Hortaçsu, 2010). Future research should also take the form of field experiments and actively manipulate the complexity of seller usernames to address the causal relation between name complexity and sale success. Field experiments may also allow overcoming the fact that our participants did not face any real (financial) risk by rating one seller higher on trustworthiness than another one, and such risk may lead to different choices/outcomes in real commercial contexts. Future experiments could also explore the effect of name complexity in the presence of other variables that influence trustworthiness in consumer domains, such as the consistency between the valences of product ratings and of the text in the written reviews about the product (Tsang and Prendergast, 2009), or the popularity of the product being sold and level of consumer Internet experience (Zhu and Zhang, 2010). Related to consumer experience in online contexts, it may be interesting to explore individuals’ experience in online marketplaces and their knowledge of how the contact with a partner develops.

The relevance of name complexity in online environments should not be limited to the case of seller usernames. Name complexity can be relevant also in the domain of the products that are transacted. In fact, pronounceability effects in other contexts have also been found with product names. For example, stock market shares with easy-to-pronounce ticker codes outperform those with difficult-to-pronounce codes (Alter and Oppenheimer, 2006), food additives with easy-to-pronounce names are considered less dangerous than their counterparts (Song and Schwarz, 2009), and pharmaceutical drugs with fluent names are perceived as less hazardous, assumed to have less side effects and increase willingness to buy (Dohle and Siegrist, 2014; Dohle and Montoya, 2017).

Another aspect of online environments in which name complexity may play a relevant role are the online platforms themselves – the websites, mobile apps, search engines, among others (Corritore et al., 2003). Because accessing the online platforms that support the commercial or social transactions is the first step to actually engage in those interactions, the name that companies or entrepreneurs choose to give to their online sites needs to be extremely well crafted, otherwise individuals may not even register with the website or download the app (Keller et al., 1998; Yorkston and Menon, 2004; Pranata et al., 2012; Kuehnhausen and Frost, 2013).

There might be, however, important exceptions to the general conclusion of our Experiments. While for most instances of online environments the general take home message of the paper is *make names short and easy*, in some contexts disfluency may be desired. Previous research shows that creating contexts where processing fluency is low increases preference for special occasion products and services (Pocheptsova et al., 2010) and perceptions of product innovativeness (Cho and Schwarz, 2006). Thus, it is likely that the effects of name complexity that we observed are different in contexts related to online privacy and security, for example (Bleha et al., 1990; Miyazaki and Fernandez, 2001; Ni et al., 2003). Although our results suggest that individuals are judged as more trustworthy when the usernames they create are short and easy-to-pronounce, the recommendations for the creation of safe passwords go exactly in the opposite direction (Gehringer, 2002; Huth et al., 2012): *make it long and difficult!* Thus, online contexts that intend to create a feeling of security and safety may gain advantage when the agents that contact with users bear complex names.

## Author Contributions

Conceptualization: RS, ST, NC, EN, and NS. Investigation: RS and NC. Data curation and analysis: RS, ST, EN, and NC. Wrote the original draft: RS, ST, NC, EN, and NS. Contributed in writing review and editing: RS, ST, NC, EN, and NS. Contributed in funding acquisition: ST and NC.

## Conflict of Interest Statement

The authors declare that the research was conducted in the absence of any commercial or financial relationships that could be construed as a potential conflict of interest.

## Footnotes

1 Specific instructions in Experiments 1–7 were: “In this study we explore how individuals form attitudes about the private sellers in Internet auction sites. You will see several screenshots of eBay sellers with some information about them. For each seller, you should indicate how trustworthy you think they are. To give your response, please use the slider that is provided bellow each seller. The scale goes from 1 to 10. The more trustworthy you think the seller is, the higher should be the number you select in the slider. Observe all the information about the seller. Pay attention to the name of the sellers, as this may play a role later in the experiment.” In each experiment, small adjustments were made to accommodate changes made to the design.

2 We also tested for gender differences in Experiments 8–10, as the experimental paradigm was largely modified, and again there were no differences between male and female participants (all *p*s > 0.470).

3 Order of the Ingroup vs. Outgroup blocks did not qualify the main effects of Pronounceability [*F*(1,73) = 3.69, *p =* 0.059], Length [*F*(1,73) = 3.10, *p* = 0.082] or Reputation (*F* < 1) on trustworthiness ratings.

4 Order of the Personal choice vs. Computer algorithm username blocks did not qualify the main effects of Pronounceability, Length or Reputation on trustworthiness ratings (*F* < 1, for all interactions).

5 An exploration of the data entering Order of the New account vs. Old accounts blocks as a between-participants factor shows that this variable qualified the main effects of Pronounceability [*F*(1,27) = 5.11, *p =* 0.032, $\eta_{p}^{2}$ = 0.16) and Length [*F*(1,27) = 7.23, *p =* 0.012, $\eta_{p}^{2}$ = 0.21]. However, these interactions show only that the differences between easy- and difficult-to-pronounce usernames follow the pattern of the main effect (easy > difficult), but the difference was larger when New accounts were shown first (*M_easy_* = 5.18, *SE* = 0.32, *M_difficult_* = 4.30, *SE* = 0.41) than second (*M_easy_* = 4.01, *SE* = 0.54, *M_difficult_* = 3.75, *SE* = 0.54). The same happens for the difference between short and long usernames (New accounts first: *M_short_* = 4.98, *SE* = 0.32, *M_long_* = 4.51, *SE* = 0.40; New accounts second: *M_short_* = 3.93, *SE* = 0.53, *M_long_* = 3.83, *SE* = 0.53). Sequence of New vs. Old accounts blocks did not qualify Reputation’s effect (*F* < 1).
