# Supplementary material for: Make It Short and Easy: Username Complexity Determines Trustworthiness Above and Beyond Objective Reputation
Source: Front Psychol. 2017 Dec 19;8:2200. doi: 10.3389/fpsyg.2017.02200 (PMC5742175; doi:10.3389/fpsyg.2017.02200)
Supplement: Supplementary file 1 [file Tables.PDF]

Supplementary Material

Make it Short and Easy: Username Complexity Determines Trustworthiness Above and Beyond Objective Reputation

Rita R. Silva*, Nina Chrobot, Eryn Newman, Norbert Schwarz, and Sascha Topolinski

* Corresponding author
Email address: rita.silva@uni-koeln.de

Table 1. Usernames used in Experiments 1 and 2

Easy (4 letters)	Medium (7 letters)	Difficult (12 letters)
Naba	Irizogh	Vlegtiqclapl
Hira	Ptonbia	Qoltdrivsgtu
Sesu	Quelizl	Cfguftakkgit
Sibo	Agruilz	Llopljuzkbqi
Rifo	Ghfinea	Mpupklzcoxik
Bulo	Sraohiq	Tqefqaycortc
Tagu	Edlokaq	Kqizutvgpapt
Kagu	Aazghik	Rfeiklxoxjkl
Fabu	Inmnaor	Yqriklbvuogt
Segu	Frhichao	Hqjthixeszquj
Madi	Bkortuj	Bmjukpolrasd
Keva	Mgtufat	Dtrguoplsmnd
Davo	Dsaergi	Efhuwopklmns
Gima	Hmiopok	Fgzutkjmnuis
Fola	Klruatg	Lkjwuiksguaa
Jela	Lghumro	Plkopuardghn
Poda	Ojhrtpe	Hguehjkopwmn
Zemi	Rijkgen	Juwtgfopkjas
Reso	Srtuhnm	Luwgthgsnmse
Saka	Zitgapk	Zhngadruismn

Table 2. Unsolvable anagrams used as usernames in Experiments 3-7

Easy-Short	Difficult-Short	Letters	Easy-Long	Difficult-Long	Letters
Batrek	eakrtb	6	fechliren	ieclhfrne	9
Galmug	uaglgm	6	lerbewnut	elnbrtweu	9
Sordot	otrsdo	6	getareigo	eoiggrtae	9
Lapnuk	unklpa	6	getelegim	eilegmtge	9
Napges	esgnpa	6	gubrevnev	evnrbvgue	9
Nerdet	erdnte	6	kelmerilo	erlmlkeoi	9
Scaztu	aztcsu	6	lehginitc	eihntclgi	9
Zetdos	eztsdo	6	neblugrob	ugnblrbeo	9
ekisepo	pskeoe	7	nimisedor	insrdmioe	9
wagratu	atrwgua	7	sorpesrop	oesrsprpo	9
lapusaw	alpwsua	7	tenkuvmur	etrmknvuu	9
lavukef	uflvkea	7	sborntrat	sobrntrta	9
knupfim	pfiumkn	7	usanitido	idnstiaou	9
rerubel	uerbrle	7	gahbufneic	aiugnhcfbe	10
pezarig	eargzpi	7	gjawusentu	eutngjwsua	10
Jaliper	ilrjpae	7	mernigwuki	ueignkrwmi	10
tahnluc	hlucnta	7	nilegituje	ignltjeieu	10
hiknilch	hhnklcii	8	tabrelchod	oacdhblrte	10
padasnut	uadnstpa	8	vagfurnesd	ausngdvfre	10
notlinfu	uotfnlin	8	beketegineb	iekbgbenete	11
gekikite	ietkkge	8	fehstoctrab	tohcbtsrcea	11
snacthec	tsrcneha	8	vanigetesch	eaintvschge	11
nekaripo	oaenpkri	8	fudnegrodan	agnrdfnduoe	11
secanamu	easuanmc	8	vijuntreber	evrbiueretjn	11
cildenug	egncldui	8	negravanlut	auegnltnvra	11

Table 3. Real eBay usernames (and pronounceability ratings) used in Experiment 8

eBay username	*M*	eBay username	*M*
drivesupply	6.46	smartechsuk	4.94
britishdeals	6.43	labazaar1509	4.54
okspots	6.41	osakaninja999	4.54
hi.entertainment	6.41	sb4100s	4.52
flythefashion	6.39	lifessw	4.52
smallelectricals	6.37	washym	4.48
buydig	6.3	mondocasawebstore	4.31
creationzonline	6.17	kunisama	4.22
monarch285	6.07	2014.takeh	4.17
virtualfoto	6.02	jebihara	4.04
digitechx	6	sacchan2008	4
imartplus	5.96	koenigsegg1115	3.8
thepcexpert24	5.85	teccoltd	3.63
allyouneedhilasstore	5.59	123cajetan	3.54
camerawestwc	5.57	noidicasa2	3.41
blaz1ngsun	5.5	elkibode	3.04
fspoint2015	5.46	torinofotoerre	2.74
ramborghini2013	5.2	eeassa	2.67
yepponshop	5.15	4kokuq	2.41
rpreporter2	5.06	nig252aheywjij	1.72

Table 4. Real names used in Experiment 9

First and last name	Pronounceability	Region
Rao Jian	Easy	Eastern Asia
Yang Dong-In	Easy	Eastern Asia
Liu Xiangying	Difficult	Eastern Asia
Kwak Dyung-Bae	Difficult	Eastern Asia
Anatoly Bashkatov	Easy	Eastern Europe
Marcin Stanowski	Easy	Eastern Europe
Nona Hubulova	Easy	Eastern Europe
Yevgeny Nazdratenko	Difficult	Eastern Europe
Krzysztof Pyziak	Difficult	Eastern Europe
Nugzar Papuashvili	Difficult	Eastern Europe
Hassan Salahi	Easy	Middle East
Yossi Farhi	Easy	Middle East
Aviran Halevi	Easy	Middle East
Seyyed Mir-Ma'soum	Difficult	Middle East
Ephraim Ben-Zvi	Difficult	Middle East
Yitzhak Almaliah	Difficult	Middle East
Amira Saleh	Easy	Northern Africa
Olusola Saanu	Easy	Northern Africa
Mofoluso Ayeni	Easy	Northern Africa
Obore Yankosor	Easy	Northern Africa
Adusei Baffour	Easy	Northern Africa
Suheir Sharawi	Difficult	Northern Africa
Oby Ezekwesili	Difficult	Northern Africa
Uche Majekodunmi	Difficult	Northern Africa
Kwadwo Mpiani	Difficult	Northern Africa
Ntiamoa Adarkwa	Difficult	Northern Africa
Jabulani Mavuso	Easy	Southeastern Africa
Motalane Monakedi	Easy	Southeastern Africa
Ndivhuwo Tshikororo	Difficult	Southeastern Africa
Mbulungeni Makungo	Difficult	Southeastern Africa
Veeran Wali	Easy	Southern Asia
Samreena Hashmi	Easy	Southern Asia

First and last name	Pronounceability	Region
Kumari Balasuriya	Easy	Southern Asia
Peshala Hansini	Easy	Southern Asia
Jyotsna Mengi	Difficult	Southern Asia
Iftikhar Lodhi	Difficult	Southern Asia
Ranjith Vitharana	Difficult	Southern Asia
Pragathi Wijetileka	Difficult	Southern Asia
Antonella Massari	Easy	Western Europe
Isabelo Herreros	Easy	Western Europe
Rudolf Ladwig	Easy	Western Europe
Gernot Nerb	Easy	Western Europe
Giulia Gortanutti	Difficult	Western Europe
Xabier Urbaneja	Difficult	Western Europe
Uwe Schütz	Difficult	Western Europe
Fritz Gelowicz	Difficult	Western Europe
