# Supplementary material for: Make It Short and Easy: Username Complexity Determines Trustworthiness Above and Beyond Objective Reputation
Source: Front Psychol. 2017 Dec 19;8:2200. doi: 10.3389/fpsyg.2017.02200 (PMC5742175; doi:10.3389/fpsyg.2017.02200)
Supplement: Supplementary file 2 [file Figures.PDF]

Supplementary Material

Make it Short and Easy: Username Complexity Determines Trustworthiness Above and Beyond Objective Reputation

Rita R. Silva[*], Nina Chrobot, Eryn Newman, Norbert Schwarz, and Sascha Topolinski

* Corresponding author
Email address: rita.silva@uni-koeln.de

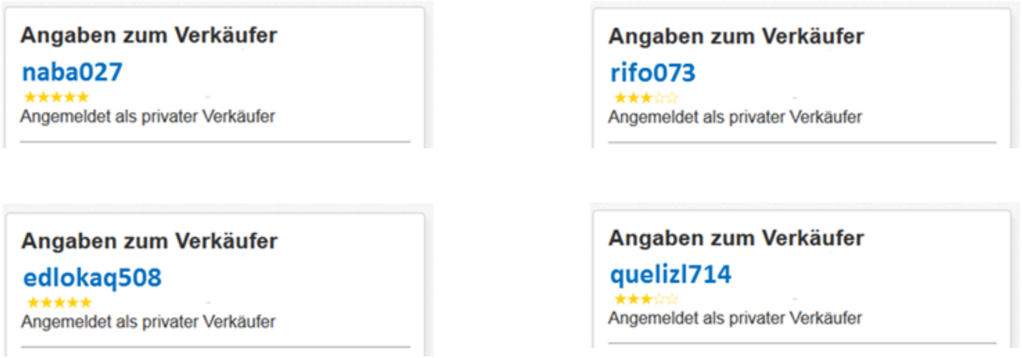

Figure 1. Examples of the profiles used in Experiments 1-7

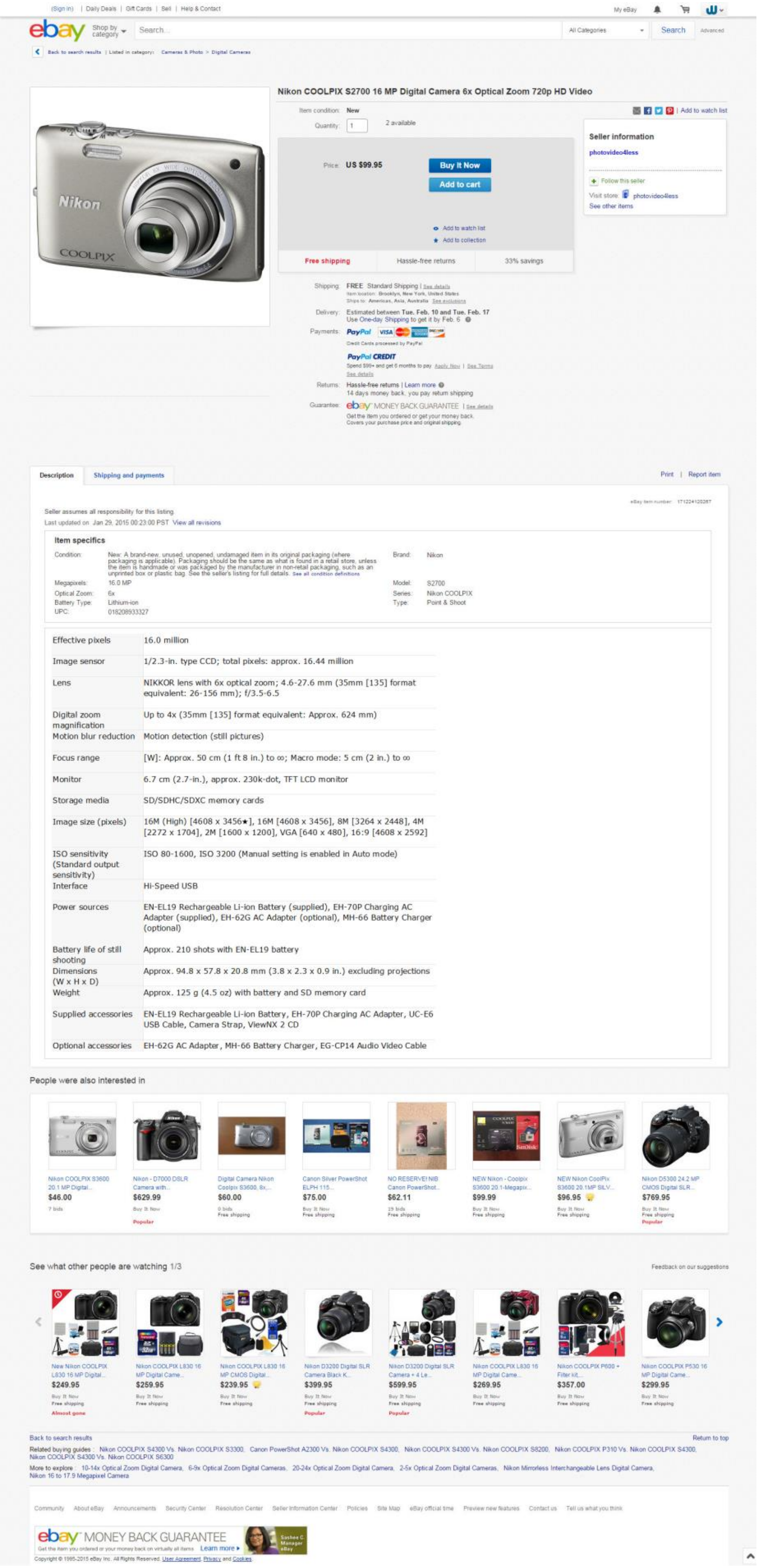

Figure 2. Product page description used in Experiment 10, in fluent version.

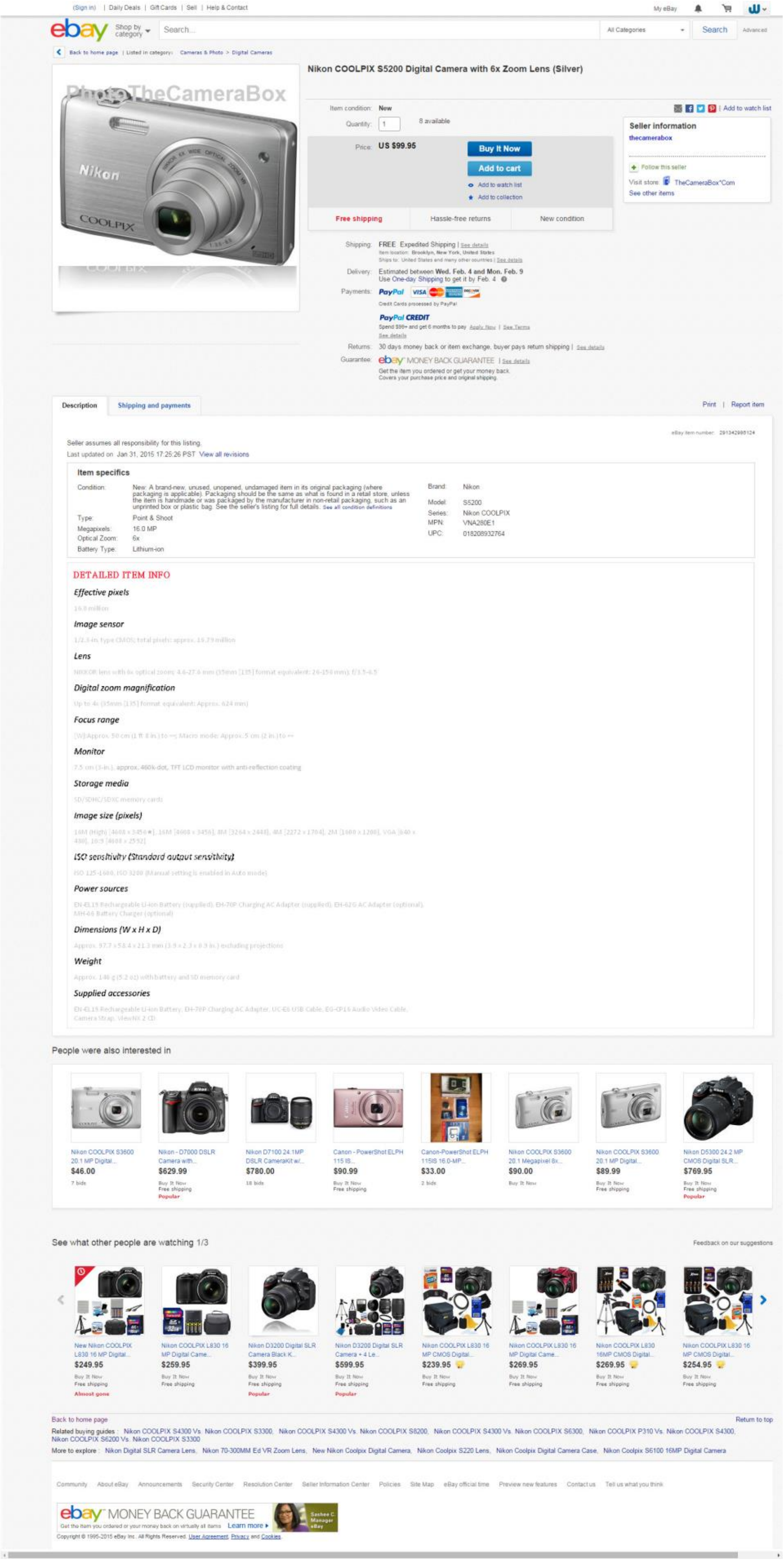

Figure 3. Product page description used in Experiment 10, in disfluent version.
